# Canopy architectural and physiological characterization of near-isogenic wheat lines differing in the *t*iller *in*hibition gene *tin*

**DOI:** 10.3389/fpls.2014.00617

**Published:** 2014-12-02

**Authors:** Carina Moeller, Jochem B. Evers, Greg Rebetzke

**Affiliations:** ^1^Tasmanian Institute of Agriculture, University of TasmaniaHobart, TAS, Australia; ^2^Centre for Crop Systems Analysis, Wageningen UniversityWageningen, Netherlands; ^3^Commonwealth Scientific and Industrial Research Organisation Plant Industry, Black Mountain LaboratoriesBlack Mountain, ACT, Australia

**Keywords:** wheat, tillering plasticity, tiller inhibition gene, *tin*, organ sizes, radiation interception, red: far-red ratio

## Abstract

Tillering is a core constituent of plant architecture, and influences light interception to affect plant and crop performance. Near-isogenic lines (NILs) varying for a *t*iller *in*hibition (*tin*) gene and representing two genetic backgrounds were investigated for tillering dynamics, organ size distribution, leaf area, light interception, red: far-red ratio, and chlorophyll content. Tillering ceased earlier in the *tin* lines to reduce the frequencies of later primary and secondary tillers compared to the free-tillering NILs, and demonstrated the genetically lower tillering plasticity of *tin*-containing lines. The distribution of organ sizes along shoots varied between NILs contrasting for *tin*. Internode elongation commenced at a lower phytomer, and the peduncle was shorter in the *tin* lines. The flag leaves of *tin* lines were larger, and the longest leaf blades were observed at higher phytomers in the *tin* than in free-tillering lines. Total leaf area was reduced in *tin* lines, and non-*tin* lines invested more leaf area at mid-canopy height. The tiller economy (ratio of seed-bearing shoots to numbers of shoots produced) was 10% greater in the *tin* lines (0.73–0.76) compared to the free-tillering sisters (0.62–0.63). At maximum tiller number, the red: far-red ratio (light quality stimulus that is thought to induce the cessation of tillering) at the plant-base was 0.18–0.22 in *tin* lines and 0.09–0.11 in free-tillering lines at levels of photosynthetic active radiation of 49–53% and 30–33%, respectively. The *tin* lines intercepted less radiation compared to their free-tillering sisters once genotypic differences in tiller numbers had established, and maintained green leaf area in the lower canopy later into the season. Greater light extinction coefficients (*k*) in *tin* lines prior to, but reduced *k* after, spike emergence indicated that differences in light interception between NILs contrasting in *tin* cannot be explained by leaf area alone but that geometric and optical canopy properties contributed. The careful characterization of specifically-developed NILs is refining the development of a physiology-based model for tillering to improve understanding of the value of architectural traits for use in cereal improvement.

## Introduction

Tillering refers to the growth of lateral shoots from axillary meristems at the plant base in Poaceae species such as wheat (*Triticum aestivum* L.) and barley (*Hordeum vulgare* L.) (Assuero and Tognetti, 2010), and is an important constituent of canopy architecture. By varying tiller number, the subsequent size and display of leaf area, and ultimately spike number, individuals in a plant stand adapt dynamically to the availability of resources chiefly light, water, and nutrients (Kirby and Faris, 1972; Schmitz and Theres, 2005; Assuero and Tognetti, 2010). Genotypic variation in the degree of tillering has been documented for different species (e.g., Richards, 1988; Dofing and Karlsson, 1993; Ishikawa et al., 2005; Borràs et al., 2009). In wheat, a North African landrace showing an uniculm phenotype was uncovered about 40 years ago (Atsmon and Jacobs, 1977). Since then, restricted tillering from this landrace has been assessed and reported to be associated with a major gene named *tin* (for *t*iller *in*hibition) mapped to the short arm of chromosome 1A (Spielmeyer and Richards, 2004). More recently, near-isogenic lines (NILs) have been developed that are genetically similar except for the presence or absence of the *tin* gene (e.g., Mitchell et al., 2012; Sadras and Rebetzke, 2013). Depending on genetic background, there are strongly restricted and semi-restricted *tin* lines producing phenotypes ranging from uniculm to bi- and oligo-culm plants (Mitchell et al., 2012, 2013). The NILs contrasting in *tin* allow new opportunities for exploring relationships between extent of tillering and canopy architecture, and provide an ideal model system as the major source for genetic differences in tillering is sufficiently known. Other characteristics of *tin* genotypes are increased seed numbers per spike, and larger, heavier seeds (Richards, 1988; Duggan et al., 2005a,b; Mitchell et al., 2012, 2013; Sadras and Rebetzke, 2013).

Detailed morphological studies on a near-isogenic pair differing in *tin* showed that axillary buds developed normally in a *tin* line strongly restricted in tillering (Kebrom et al., 2012). However, the outgrowth of tiller buds during the transition of the main shoot apex from vegetative to reproductive growth ceased earlier in the *tin* than in the free-tillering NIL (Kebrom et al., 2012). As a consequence, the *tin* line produced fewer tillers than its free-tillering sibling. The mechanisms controlling bud outgrowth in *tin*-containing genotypes are arguably not well understood. It has been observed that the basal internodes in the strongly restricted *tin* line, which were solid rather than hollow, elongate earlier compared to the free-tillering line. This has been hypothesized to divert sucrose away from developing tiller buds arresting bud growth (Kebrom et al., 2012). However, the magnitude of the suppression of tiller bud outgrowth varies between *tin*-containing lines suggesting that there are interactions between *tin* and other genes to affect final tiller number (Richards, 1988; Mitchell et al., 2013).

Under conditions favorable for growth, there is close synchrony between tiller and leaf appearance, and the first tiller typically appears when the third leave on the main shoot is fully expanded (Klepper et al., 1983; Kirby and Appleyard, 1987). There is large phenotypic plasticity in maximum tiller number, which is modulated by environmental cues including shading, and the availability of water and nutrients (Sharma, 1995; Prystupa et al., 2003; Sadras and Slafer, 2012; Allard et al., 2013). In addition to competition for resources, changes in light quality, represented by the ratio between red and far-red light intensity (R:FR), sensed by phytochrome at the plant base have been proposed to induce the cessation of tillering (Smith et al., 1990; Davis and Simmons, 1994). A lowered R:FR in the light reflected from neighboring vegetation acts as an early warning signal for future competition before photo-assimilates become scarce (Casal et al., 1986; Franklin and Whitelam, 2005). In free-tillering (non-*tin*) wheat grown at varying plant densities, Evers et al. (2006) showed that tillering ceased consistently when R:FR dropped below 0.35–0.4 and levels of photosynthetic-active radiation intercepted by the canopy exceeded 40–45%. It is unknown if tillering in *tin* genotypes responds to an R:FR signal, and if there are differences in the response to R:FR between NILs contrasting for *tin*.

Following the cessation of tillering, shoot numbers decline to varying degrees until anthesis and sometimes soon after (Kirby and Faris, 1972; García del Moral and García del Moral, 1995). Tillers die as a consequence of plant internal competition for limited resources with allocation directed firstly to the main shoot at the expense of the phenologically younger primary and higher-order tillers (Lauer and Simmons, 1988). In wheat, up to 60% of tillers can abort and die under normal field conditions (Stapper and Fischer, 1990; Sharma, 1995; Berry et al., 2003; Duggan et al., 2005b). Tiller mortality has been associated with a net loss of dry matter as there is no net redistribution of assimilate from non-surviving shoots (Berry et al., 2003), and is often negatively correlated with seed yield (Sharma, 1995; Berry et al., 2003). It has been argued that increasing the ratio of productive, seed-bearing tillers to the maximum number of tillers (tiller economy) can improve resource-use efficiency to increase crop yields (Richards, 1988; Sharma, 1995; Kebrom et al., 2012). Calculated from data of Mitchell et al. (2013), the tiller economy of five *tin*-containing NILs was +8% under mild and +16% under severe terminal water-stress, but only +3% when the *tin* lines were irrigated. However, the association between reduced tillering, greater tiller economy and yield is not clear cut. When Mitchell et al. (2013) compared the NILs under mild water-stress, strongly restricted *tin* lines increased seed yield by 11% while semi-restricted *tin* lines decreased yield by 15%. Under severe water-stress, both reduced- and free-tillering NILs produced similar yields. In irrigated environments, the seed yield of *tin*-containing lines was reduced by 9 to 24% (Duggan et al., 2005a; Mitchell et al., 2013), and hence the plant's ability to respond more plastically to resources supply was advantageous.

Lower tiller numbers in *tin*-containing lines would influence the subsequent size and distribution of leaf area within the plant, and the vertical distribution and interception of radiation by the canopy. Contrasts in light levels in a plant stand caused by mutual shading of individual leaves or incomplete ground-cover at maximum canopy expansion can restrain growth and ultimately yield (Duvick, 2005; Amthor, 2010). However, canopy architecture has remained an under-explored trait in plant breeding despite that changes to architectural attributes such as the sizes, number, and display of leaves could improve the efficiency of radiation interception and therefore plant performance (Long et al., 2006). This may be partly because detailed measurements of architectural attributes are time consuming and tedious, and partly because architecture is only one of many constituent traits of seed yield. Architectural differences between NILs contrasting in *tin* have not been quantified in detail; however, Richards (1988) reported that the area of the flag and penultimate leaf was greater in *tin* genotypes compared to free-tillering lines. Duggan et al. (2005b) found similar leaf area indices (LAI; unit leaf area per unit ground area) and light interception in lines contrasting for *tin*, and suggested that larger leaves in *tin* genotypes may compensate for an overall reduction in leaf number per plant. Mitchell et al. (2013) reported that strongly restricted *tin* lines produced lower LAIs and intercepted less radiation, while semi-restricted *tin* lines performed similar to their free-tillering sisters.

The NILs contrasting for *tin* provide a unique model system to explore relationships between tillering, canopy architecture, and resource capture. The current study aims to explore the architectural basis for differences in light capture in NILs contrasting for *tin* and representing two contrasting genetic backgrounds. The relationships were assessed in terms of leaf and tiller appearance, sizes of fully grown organs, and light quality and quantity. This study is a first step in the development of an architectural model (Vos et al., 2010; Evers and Vos, 2013) of *tin* phenotypes—a tool that could assist in the identification of desirable plant architectural traits for use in trait-based selection (Long et al., 2006; Richards et al., 2010; Rebetzke et al., 2013).

## Methods and materials

### Site and genotypes

Experiments with two pairs of NILs contrasting for the *tin* gene (B_tin_ and B; 7770_tin_ and 7770) were conducted in 2012 and 2013 at experimental sites in Canberra, Australia (35.20°S, 149.08°E). The location is characterized by a semi-arid moisture regime (Williams et al., 2002), and has an average annual rainfall of around 616 mm, and average annual minimum and maximum temperatures of 6.5°C and 19.7°C, respectively (Bureau of Meteorology, 2014). The source of *tin* in line B_tin_ was the Israeli uniculm line 492 (Atsmon et al., 1986). The NILs B_tin_ and B (also referred to as B+ and B- in literature) have been used in different studies (e.g., Richards, 1988; Kebrom et al., 2012), and were obtained from a population developed by crossing line 492 to the Australian cultivar Banks with subsequent backcrossing to generate a BC_5_-derived line that was homozygous for *tin*. The source of *tin* in line 7770_tin_ was the CSIRO line CS971. The NILs 7770_tin_ and 7770 (also referred to as 7770P and 7770N in literature) were developed by crossing a Silverstar-based, *tin*-containing NIL to the cultivar Wyalkatchem, and subsequent backcrossing to Wyalkatchem before inbreeding to produce BC_1_F_5:6_ plants heterozygous for *tin*. These plants were then self-pollinated to develop a pair of lines near-isogenic for *tin*. The presence of *tin* was confirmed by genotyping with a tightly linked wheat SSR marker *gwm136* (Spielmeyer and Richards, 2004).

### Experimental conditions

In 2012, wheat was sown after the winter frost period (13/09) in the field at the Ginninderra experiment station, Canberra. The row spacing was 0.2 m and the sowing depth 0.05 m. Fertilizer nitrogen (N) was drilled into the soil at planting at a rate of 25 kg N/ha. A second rate of 15 kg N/ha was broadcast at early stem elongation (29/10). Fungicide (Prosaro®) was sprayed twice to prevent diseases. The genotypes were grown at two plant densities in irrigated and rain-fed environments in a randomized block design with three replications per treatment combination. Irrigation was applied to the irrigated block on 30/10 (25 mm) and 22/11 (20 mm). The size of individual plots was 5 m^2^. The stands were thinned when plants had about three to four leaves to achieve a low (LD; target plant density: 150 plants/m^2^) and high (HD; target: 250 plants/m^2^) plant density treatment.

In 2013, wheat was hand-sown in autumn (21/05) into raised beds located in a netted enclosure at Black Mountain, Canberra. Levels of photosynthetically active radiation were 13% lower in the enclosure than outside. The soil in the raised beds (0.2 m height) consisted 60% of a compost-based potting mix and 40% loam. Nutrients were supplied in the soil mix at rates of about 17 kg N/ha, 8 kg P/ha, and 6 kg K/ha. Raised beds were formed 1 week before sowing over soil. The lines were sown at 0.05 m depth, and 0.2 m between-row and 0.04 m within-row spacing. When plants had three leaves, any gaps in the stand were filled by replanting individuals of identical phenological age. Thus, each line grew at a uniform density of 125 plants/m^2^. Irrigation was applied up to flowering to avoid water-stress (22/05: 10 mm; 12/07: 50 mm; 11/09: 10 mm; 25/09: 5 mm; 5/10: 10 mm). Fertilizer N was applied at rates of 30 (12/07), 20 (29/07), and 40 (23/08) kg N/ha. Fungicide was applied as required. The size of individual plots was 2.2 m^2^. There were two replications per line in a completely randomized design.

### Sampling

The minimum width of the border excluded from sampling was 0.4 m in 2012 and 0.2 m in 2013. In 2012, plant establishment was assessed for each plot by counting plants in 1 m row when plants had about 3–4 leaves, while 125 plants/m^2^ were established in 2013 as described above. At crop maturity, three rows of 0.4 m length were sampled to assess the number of spikes/m^2^, and the average height of the crop stand was measured.

### Phenology and tiller identification

The Zadoks et al. (1974) decimal code (Z-score) for growth stages of cereals was adopted to record leaf numbers, inflorescence emergence (Z49-Z59), anthesis (Z60-Z69), and the start of seed filling (Z70). For example, a leaf Z-score of Z13 indicated three unfolded, mature leaves on a shoot, and Z13.5 indicated three unfolded, mature leaves with the size of the emerging fourth leaf being 50% of that of the third leaf. The emergence of the inflorescence was scored with Z49 indicating “first awns visible,” Z55 “50% of inflorescence emerged,” and Z59 “emergence of inflorescence completed.” The anthesis score Z60 indicated “start of anthesis” (i.e., anthers released on about 5% of spikes in a plot), and Z65 “anthesis half-way” (i.e., anthers released on 50% of spikes in a plot).

**Figure 1:**
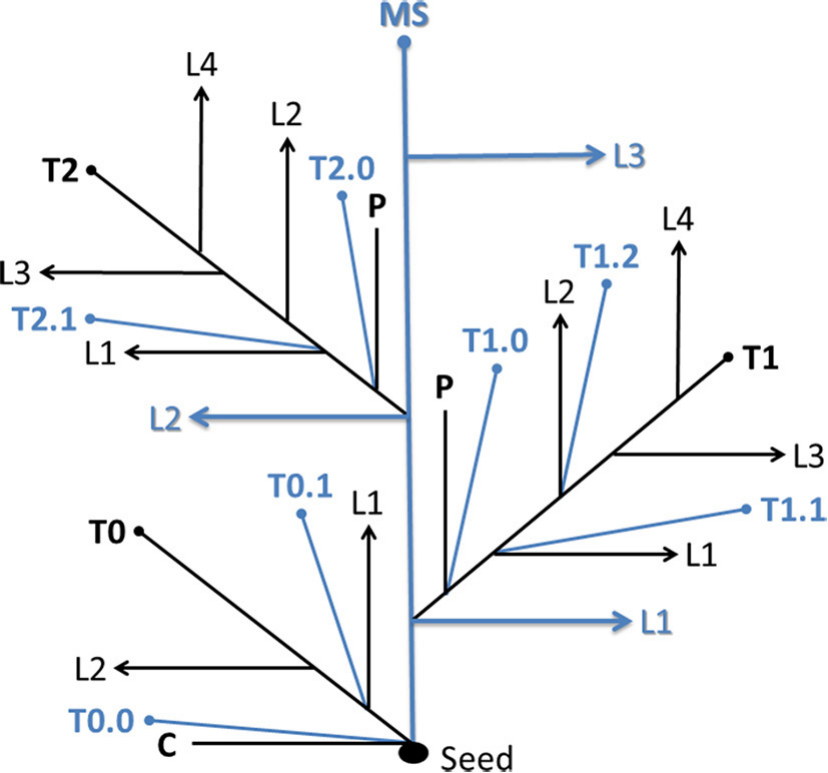
**System for naming tiller types**. Detailed explanations are given in text. MS, main shoot; T, tiller; L, leaf; C, coleoptile; P, prophyll.

Tillers were identified with reference to the leaf, prophyll, or coleoptile axil in which they appeared (Klepper et al., 1983; Kirby and Appleyard, 1987; Bos and Neuteboom, 1998). Briefly, tillers growing from the main shoot (MS) are primary tillers, and those growing from primary tillers are secondary tillers, etc. The 1st (2nd, 3rd, etc.) primary tiller emerging from the leaf axil of the 1st (2nd, 3rd, etc.) leaf of the main shoots is identified as T1 (T2, T2, etc.). The secondary tiller emerging from the leaf axil of the 1st leaf of T1 (T2, T3, etc.) is labeled T1.1 (T2.1, T3.1, etc.). The coleoptile tiller is named T0. Secondary tillers emerging from the prophyll (modified leaf similar to the coleoptile) of a parent shoot are named T1.0, T2.0, etc. (Figure 1).

To identify tiller presence, plants were sampled two times in 2012 (22/10 and 23/10; *n* = 10 plants) and three times in 2013 (13/09, 05/10, and 23/10; *n* = 20–25 plants). At each occasion, the presence of a specific tiller (alive and senescing) was noted. In 2013, changes in shoot number over time were monitored on an area basis (two adjacent rows with eight plants each) by counting the number of green shoots on the same plants every week up until the end of stem elongation.

### Organ sizes

The sizes of fully grown plant organs (width and length of leaf blades, length of leaf sheaths, internode and peduncle length) were estimated for each phytomer and shoot type (i.e., MS, T1, T2, etc.). A phytomer consists of an internode, a node above the internode, a sheath growing from the node, and a leaf blade (Forster et al., 2007; Vos et al., 2010). Phytomers are counted acropetally on each shoot type. For example, the 8th leaf of the MS (T1, T2, etc.) belongs to phytomer eight of that shoot.

To estimate organ sizes, destructive samples were taken two times in 2012 (22/10 and 28/11) and three times in 2013 (13/08, 15/09, and 23/10). On each occasion in 2012, 3–4 plants were collected at random from all plot per treatment. In 2013, 6–8 plants were sampled at random from two plots per treatment. The internode length data were used to calculate the phytomer shift values for each shoot type and derive the relative phytomer number (RPN; Evers et al., 2005; Vos et al., 2010). To obtain the RPN, a phytomer shift value, which is characteristic for each tiller, is added to the phytomer number (PN), the result is the superposition of tiller and main shoot data. The shift value for the main shoot is zero (RPN = PN). The shift values for each tiller type were calculated by simultaneously (i) fitting a linear regression model to the data of main shoot internode length data vs. PN, and (ii) minimizing the distance between the tiller internode data and the linear model. This was done by finding values for the tiller shifts and the parameters of the linear model such that the root-mean-squared error (RMSE) was minimized using the solver add-in of Microsoft Excel 2010. The frequency of occurrence of higher-order tillers was commonly low resulting in low numbers of replications (*n* = 1–2). In these cases the data were excluded from the analysis.

### Green leaf area

To estimate the Leaf Area Index (LAI; leaf area per unit ground area), the area of green leaf blades was measured with a leaf area meter (Delta-T-Devices Ltd., Burwell, UK). In 2012, samples were taken at anthesis (25/11) from 0.4 m^2^ of which a subsample of 10 plants was used to determine LAI. In 2013, to obtain a better understanding of the vertical distribution of leaf area, stratified cuts were taken *in-situ* at the start of spike emergence (19–30/09) and at flowering (15–18/10). At each occasion, 2 × 8 plants (0.13 m^2^) per plot were sampled. Stratified clips were taken at 0–0.15 m (ground-level), 0.15–0.3 m, 0.30–0.45 m, 0.45–0.6 m, 0.6–0.75 m, and, depending on final canopy height, 0.75–0.9 m to estimate the LAI for each canopy layer.

### Ground-cover

The percentage ground-cover (GC%) was estimated from digital camera photos taken during tillering. The software Canopy Cover was used to convert green pixels into GC% (Li et al., 2010).

### Light interception

Levels of Photosynthetic Active Radiation (PAR, μmol/m^2^/s) above (I_0_) and below (I_1_) the crop canopy (0–0.05 m above ground-level) were measured within 3 h around noon using a ceptometer (AccuPAR PAR-80, Decagon Devices Inc., Pullman WA, US). The data were used to estimate the percentage PAR (PAR% = I_1_ / I_0_×100) and the percentage intercepted PAR (IPAR% = I_0_ − I_1_ / I_0_×100). Measurements were taken up until anthesis; five times in 2012 and 17 times in 2013. In 2013, stratified measurements of PAR (two replications per treatment) were taken six times between the appearance of the flag leave and the start of seed-filling.

To relate light interception to leaf area, stratified measurements were taken at the same heights as for the estimation of LAI: 0–0.15 m, 0.15–0.3 m, 0.30–0.45 m, 0.45–0.6 m, 0.6–0.75 m, 0.75–0.9 m, and above the canopy. To describe the light interception characteristics of NILs differing in *tin*, Monsi and Saeki's (1953) modified version of the Lambert-Beer law was used:

$${\text{I}}_{1}={\text{I}}_{0}\times \exp(\text{​}-k\times {\text{LAI}}_{1})$$

Where I_1_ is the radiation received at a specific canopy height, I_0_ is the incident PAR% (typically 100%), *k* is the light extinction coefficient of the canopy, and LAI_1_ is the leaf area index at the height of I_1_.

### Red to far-red ratio

In 2013, R:FR was measured at around noon at the plant base between tillering (weekly measurements) and the start of flowering using a Skye SKR100/116 fiber optic probe with diffuser (Skye Instruments Ltd, Llandrindod Wells, UK). The sensor with diffuser accepts light from 180° hemisphere, while a narrower field of view of 80° hemisphere is measured by sensors without diffuser (e.g., Evers et al., 2006). During the measurements, the sensor faced away from the sun (south in the southern hemisphere), and parallel to the soil surface and green shoot structures. At each occasion, five measurements were taken in each treatment.

### Chlorophyll status

A chlorophyll meter (SPAD 502, Spectrum Technologies Inc., Aurora, IL, US) was used to estimate the leaf chlorophyll status (Chl). Measurements were taken about 40 mm from the leaf tip on 4–6 leaves of the same physiological age. In the 2012, measurements were taken on flag leaves of main shoots and subjacent 4th leaves on 14/11 (flag leaves fully expanded). In 2013, Chl was measured on 13–17/09 (mid/late booting stage), 05/10 (~start of anthesis) and 23/10 (seed-filling) on flag leaves and all subjacent alive leaves on main shoots and primary tillers T1 and T2.

### Weather data and calculations

Daily minimum and maximum temperatures (°C), and solar radiation (MJ m^2^/day) were sourced from the Ginninderra experiment station (35.20°S, 149.08°E, 600 m a.s.l). The weather station was located close to the experiment in 2012, and about 6 km from the Black Mountain site used in 2013. In 2013, netting of the enclosure reduced radiation by 13% and the solar radiation data were adjusted accordingly. Daily rainfall (mm) data were recorded close to the experiments.

Cumulative thermal time (cTT in degree days, °Cd) was calculated from emergence as

$$\text{cTT}{(}^{{}^{\circ}}\text{Cd})=\sum_{i\;=\;1}^{\text{n}}\left({\text{T}}_{\text{max}}+{\text{T}}_{\text{min}\_\text{b}}\right)/2$$

Where T_max_ is the mean maximum temperature, T_min_b_ is the mean minimum temperature adjusted for a base temperature at which development is thought to stop, and n is the number of days of temperature observations used in the summation (Ritchie and NeSmith, 1991). The base temperature was 0.0°C, i.e., if the mean minimum temperature was lower than the base temperature the consequent value of T_min_b_ was zero on that day (Ritchie, 1991). The phyllochron, defined as the thermal time interval between the appearances of successive leaves on a shoot, was obtained as the slope of the linear regression of cTT against the number of leaves with the intercept set to zero.

### Data analysis

Seasons were analyzed separately in GenStat (16th Edition, VSN International, Hemel Hempstead, UK). For the analysis of the 2012 data (except organ sizes and tiller types), mixed models were fitted using ASREML. The best spatial model was determined statistically after sequential model fitting and included the factors of the experimental design (“environment,” “genotype,” and “plant density”). The residual variation was modeled with autoregressive (AR1) row and column terms, and fitting of a linear row term (all effects statistically different from zero at *p* = 0.05). The interaction and main effects of “environment” (irrigated and rain-fed) were non-significant (*p* > 0.70), and the effects were subsequently pooled with the residual. Other data were analyzed using the GLM procedure in GenStat. The 2012 (factors: “genotype” and “density”) and 2013 (factor: “genotype”) data on presence/absence of tiller types were transformed using an arcsine square root transformation before the statistical analysis.

## Results

### Seasonal conditions and phenology

**Figure 2:**
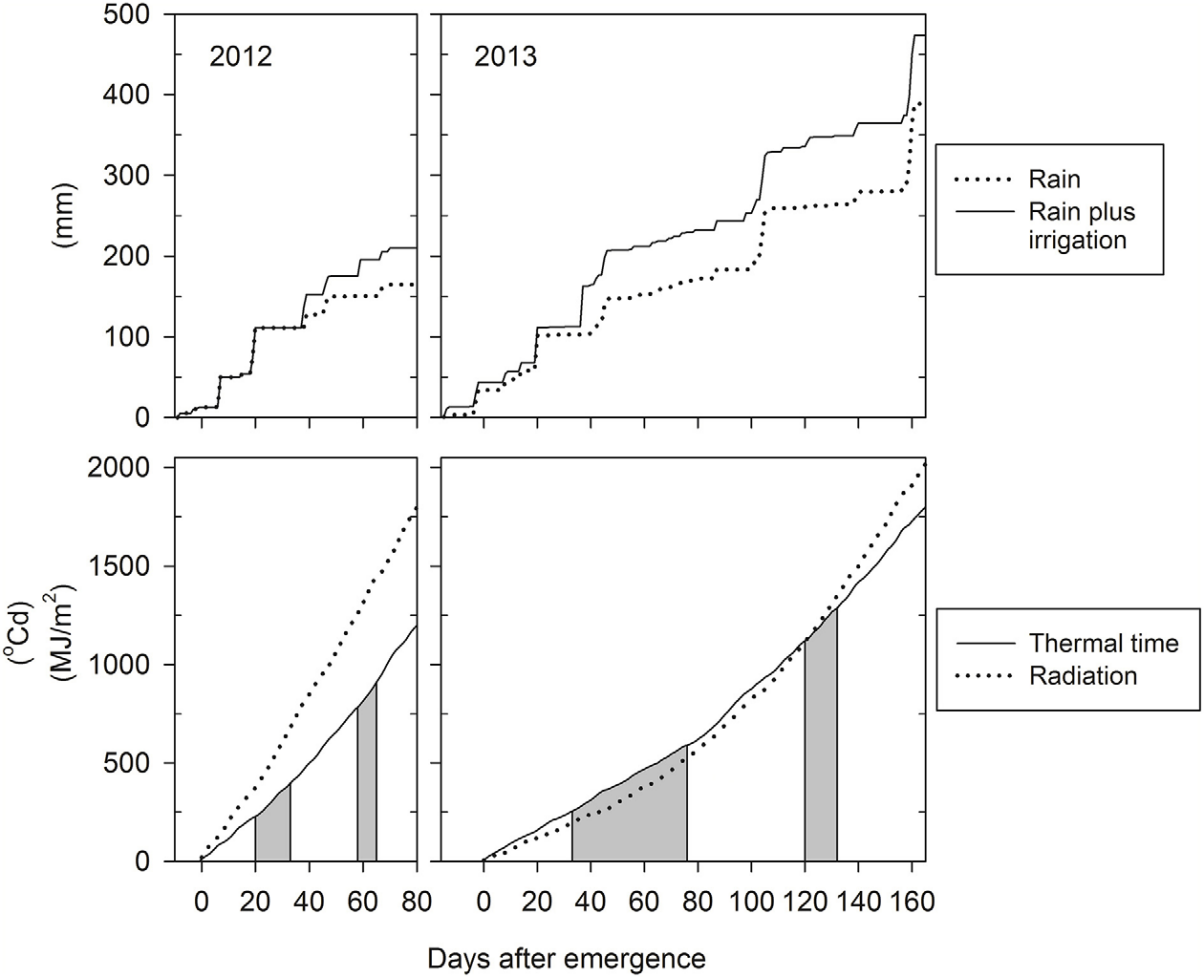
**Seasonal growth conditions in 2012 and 2013: cumulative water supply by in-crop rainfall plus irrigation (upper panels), and cumulative thermal time and solar radiation from emergence (22/09/2012 and 05/06/2013) to maturity (lower panels)**. The shaded areas indicate the tillering and flowering windows, respectively.

Contrasting photo-thermal conditions and levels of water supply (Figure 2) resulted in lifecycle durations of only ~90 days in 2012 but ~180 days in 2013, i.e., plant development was hastened in 2012 compared to 2013. In 2012, late-sown wheat experienced increasing day lengths and temperatures from emergence onward. In-crop water supply (rainfall plus irrigation from sowing until maturity) was 200 mm only, and symptoms of water-stress became apparent from around tillering onward. In contrast, day-lengths and temperatures decreased initially in 2013, and in-crop water supply was 475 mm. Tillering commenced at 229°Cd (20 days after emergence, DAE) in 2012 and at 256°Cd (33 DAE) in 2013. Anthesis commenced earlier in 2012 compared to 2013 both in terms of the number of days required from emergence to start of anthesis (61 vs. 122 DAE) and the average amount of thermal units accumulated over this period (826 vs. 1143°Cd). Each near-isogenic pair had similar anthesis dates (Table 1), which is important for any comparisons to be unconfounded by differences in phenology. The pair 7770/7770_tin_ flowered 3–5 days earlier than the pair B/B_tin_ consistent with the earlier maturity of the parental donor Wyalkatchem (Table 1). Across genotypes, the average phyllochron was 76°Cd in 2012 and 87°Cd in 2013, which corresponds to a difference in leaf appearance rate of about 1 day. Some of this difference could be related to the source of temperature data as described above. However, the faster leaf appearance in 2012 is in line with an overall hastened plant development in this short season, where plants produced about two main stems leaves less compared to 2013 (Table 1).

**Table 1:** **Estimated date of start of anthesis (Z60), days after emergence (DAE) to Z60, cumulative thermal time (cTT) from emergence to Z60, average maximum number of main shoot leaves (LN), and phyllochrons of main shoot leaves of near-isogenic wheat lines differing in the *tin* gene grown in two contrasting seasons at Canberra**.

**Line**	**7770**		**7770_**tin**_**		**B**		**B_**tin**_**	
**Season**	**2012**	**2013**	**2012**	**2013**	**2012**	**2013**	**2012**	**2013**
Anthesis (Z60)^a^	19/11	03/10	19/11	04/10	24/11	06/10	24/11	06/10
DAE	58	120	58	121	63	123	63	123
r^2^	0.89	0.78	0.89	0.85	0.86	0.91	0.78	0.95
RMSE (days)	1.5	3.9	1.6	2.6	1.6	2.6	1.4	1.9
cTT at Z60 (°Cd)	784	1121	784	1131	868	1160	868	1160
LN	8.1	9.9	8	10	8.3	10	8.5	10.1
s.e.	0.22	0.07	0.0	0.0	0.14	0.07	0.16	0.07
Phyllochron (°Cd)^b^	77	85	73	85	79	90	75	86
r^2^	0.90	0.95	0.87	0.95	0.93	0.95	0.94	0.95
RMSE	44	62	45	60	36	62	36	63

a *Start of anthesis (Z60) date = a × date + b; linear regression of observed Z-scores (Z50-Z70) against date*.

b *cTT = a × LN, with a corresponding to the phyllochron*.

### Architectural attributes: organ sizes

**Figure 3:**
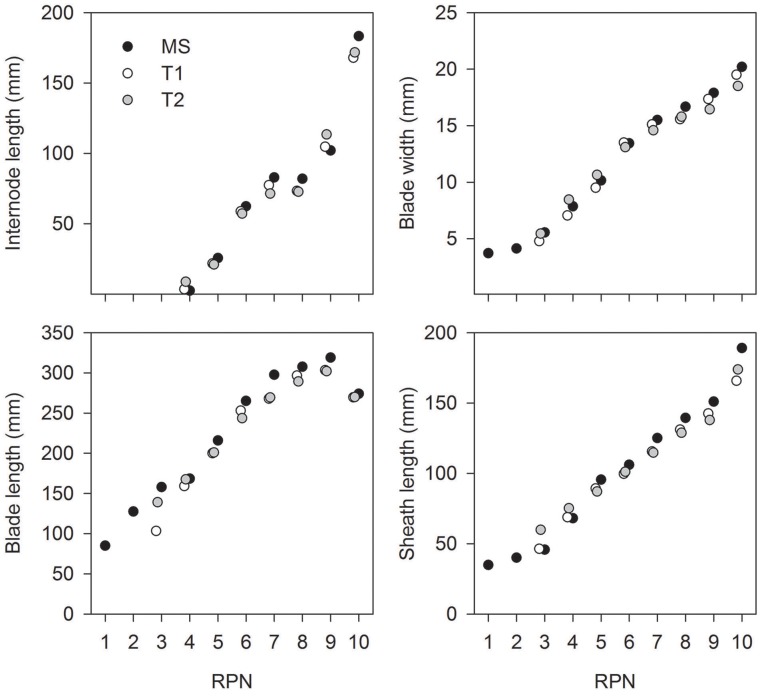
**Average size of plant organs against relative phytomer number (RPN) for the main shoot (MS) and primary tillers T1 and T2 of line B_tin_ grown at Canberra, 2013**.

In each genotype, the distribution of organ sizes along a shoot (i.e., the gradual changes in blade width and length, sheath length, and internode length with phytomer number) was similar for main shoots and tillers. This can be demonstrated by applying the concept of Relative Phytomer Number (RPN; Figure 3). For example, the RPN for T1 of line B_tin_ was “RPN= phytomer number + 1.81” and that for T2 was “RPN = phytomer number + 1.86” (RMSE = 15.6; Figure 3). These similarities were greater in 2013 (long season and minimal moisture stress) compared to 2012 (short season and severe water-stress) (data not shown). Because of the architectural similarities between main shoots and tillers, only MS data are presented in the following sections.

**Figure 4:**
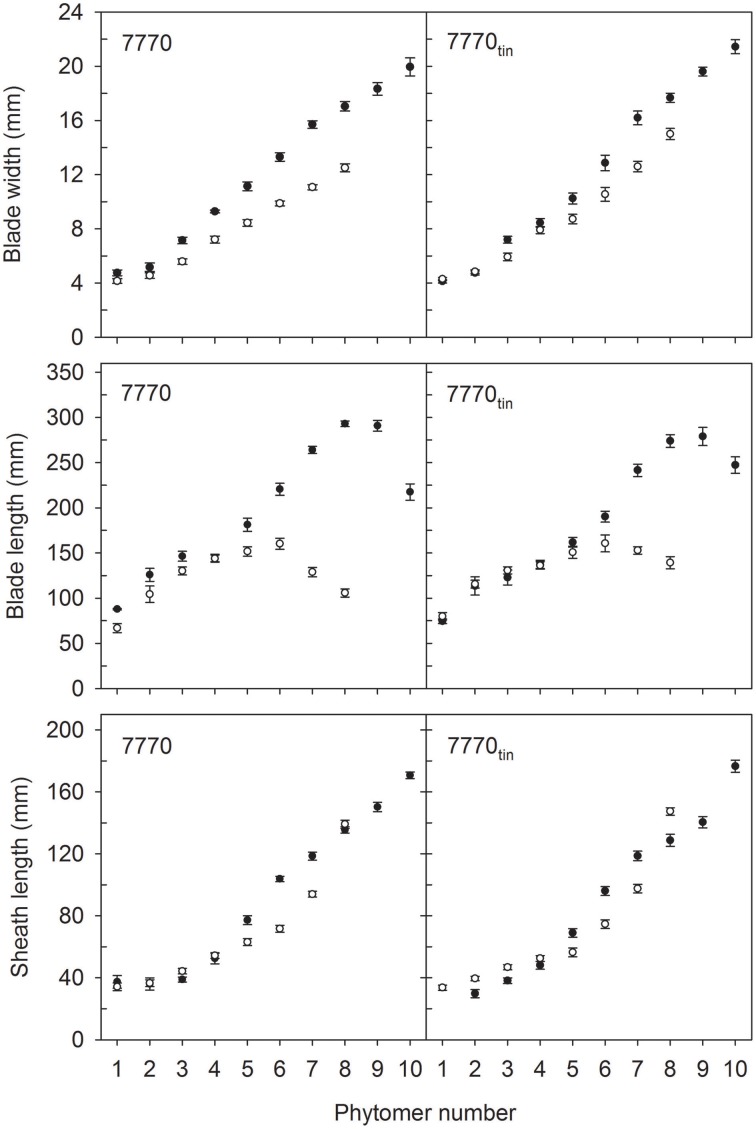
**Characteristics of main shoot leaves in lines 7770 and 7770_tin_, which are near-isogenic for the *tin* gene, grown in 2012 (white symbols) and 2013 (black symbols) at Canberra**. Error bars show ± one standard error. Only data of plants with seven (2012) and 10 main shoot phytomers (2013) are shown.

**Figure 5:**
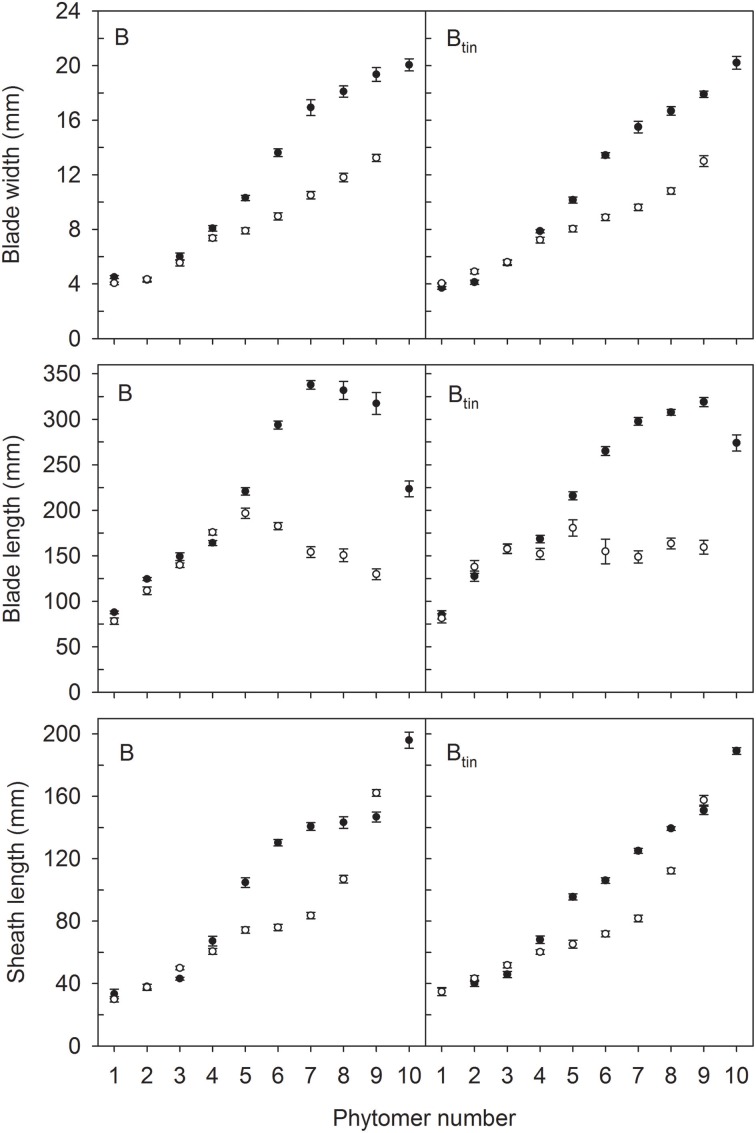
**Characteristics of main shoot leaves in lines B and B_tin_, which are near-isogenic for the *tin* gene, grown in 2012 (white symbols) and 2013 (black symbols) at Canberra**. Error bars show ± one standard error. Only data of plants with nine (2012) and 10 main shoot phytomers (2013) are shown.

The distribution of leaf sizes along the main shoot varied between *tin* and non-*tin* genotypes in both seasons (Figures 4, 5). The main features were the larger flag leaves of *tin* lines, and that the longest leaf blades were observed at lower phytomers in the free-tillering lines compared to the *tin* lines (Figures 4, 5).

In 2012, organ sizes were similar at both plant densities (*p* > 0.60) despite significant differences between densities in the number of plants at establishment (*p* < 0.001), and the number of shoots measured at flag leaf expansion (*p* = 0.023). Thus, the effect of density on organ sizes was pooled with the residual variation. More specifically, the length of flag leaves was 105 and 139 mm in lines 7770 and 7770_tin_, and 130 and 159 mm in lines B and B_tin_ (*p* < 0.001, LSD = 20). The width of flag leaf blades was 12.5 and 15 mm in lines 7770 and 7770_*tin*_, and about 13 mm in B and B_*tin*_ (*p* < 0.001, LSD = 1.1). Similarly, the penultimate leaf was larger in the *tin* lines for both blade width (*p* < 0.001, LSD = 0.8) and length (*p* < 0.001, LSD = 19). The longest leaf blades were observed at about phytomer five in 2012. Here, leaf blades were significantly longer in the free-tillering compared to the *tin* lines (*p* < 0.001, LSD = 17), while the blade widths were similar (*p* = 0.16) (Figures 4, 5).

In 2013, the length of flag leaf blades was 217 and 247 mm in lines 7770 and 7770_tin_, and 224 and 274 mm in lines B and B_tin_ (*p* < 0.001, LSD = 26), while there were no differences in flag leaf width, which was about 20 mm in all lines (*p* = 0.2). There was a significant effect of genotype on the width (*p* = 0.017, LSD = 1.12) and length (*p* = 0.006, LSD = 25) of the penultimate leaf though this was unrelated to the presence or absence of the *tin* gene. Similar to the 2012 season, the longest leaf blades were observed at lower phytomers in free-tillering lines compared to the *tin* lines (7–8 vs. 9). At phytomer eight, for example, leaf blades were significantly longer in the free-tillering lines (*p* < 0.001, LSD = 18) while leaf blades were wider in the *tin* lines (*p* = 0.039, LSD = 1) (Figures 4, 5).

**Figure 6:**
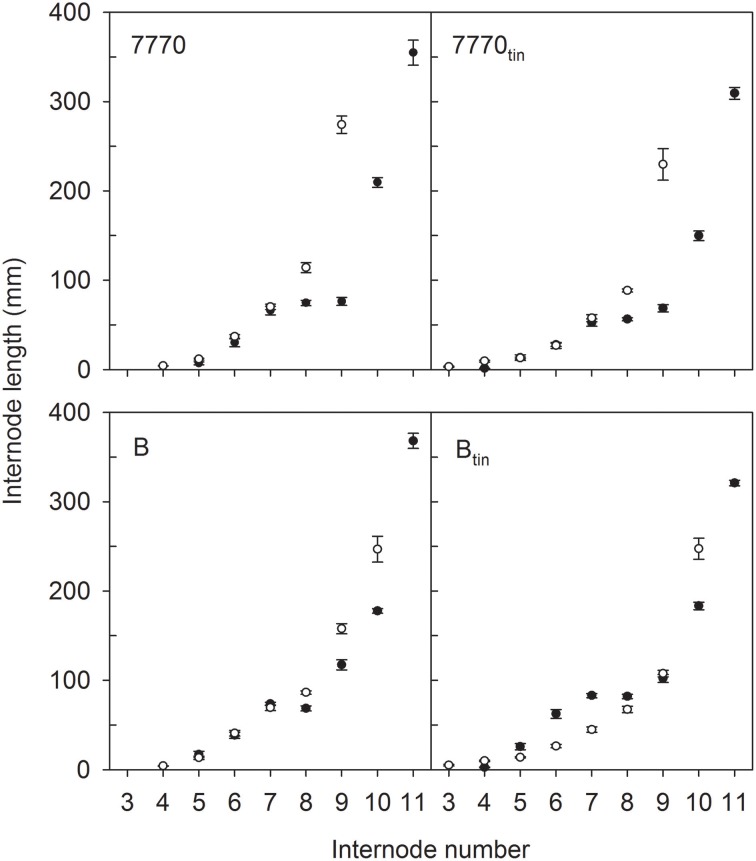
**Internode lengths of two pairs of near-isogenic lines differing in the *tin* gene (7770/7770_tin_ and B/B_tin_) grown at Canberra in 2012 (white symbols) and 2013 (black symbols)**. The values at the highest internode number represent the peduncle length, and numbers of subjacent internodes correspond to the respective phytomer numbers. Error bars show ± one standard error.

The pattern of fully elongated internodes varied between *tin* and non-*tin* genotypes (Figure 6). For example, internode elongation commenced at a lower phytomer in the *tin* lines. In 2012, the 1st and 2nd basal internode did not elongate in any line while the final length of the 3rd basal internode (phytomer 3) was 3 mm in line 7770_tin_ and 5 mm in line B_tin_ but 1 mm in the free-tillering NILs (*p* < 0.001, LSD = 2). In 2013, the 1st, 2nd, and 3rd basal internode did not elongate in any line. The final length of the 4th internode (phytomer 4) was 2.5 mm in line B_tin_ and 1 mm line B, and about similar in the pair 7770_tin_ and 7770 (1.3 vs. 1 mm; *p* = 0.01, LSD = 0.6).

The peduncle and the internode below the peduncle tended to be shorter in the *tin* compared to the free-tillering lines (Figure 6). In 2012, the length of the internode below the peduncles was 114 and 87 mm in lines 7770 and 7770_tin_, and 158 and 108 mm in lines B and B_tin_ (*p* < 0.001, LSD = 14), while peduncles tended to be shorter in the *tin* lines although the differences were only significant at *p* = 0.16. In 2013, the peduncle was significantly shorter in the *tin* lines (*p* < 0.001), i.e., peduncle length was 355 and 309 mm in lines 7770 and 7770_tin_, and 368 and 321 mm in lines B and B_tin_ (LSD = 27). The length of the internode subjacent to the peduncle was 210 and 150 mm in lines 7770 and 7770_tin_ but was similar in lines B (178 mm) and B_tin_ (183 mm) (*p* < 0.001, LSD = 13).

Genotypic differences in internode lengths resulted in different canopy heights. In 2012, the final height of the canopy was shorter in the *tin* than in the free-tillering lines, i.e., 0.64 m and 0.55 m in lines 7770 and 7770_tin_, and 0.69 m and 0.65 m in lines B and B_tin_, respectively (*p* < 0.001, LSD = 0.033). In 2013, canopy height was again shorter in the *tin* line of the pair 7770_tin_ and 7770 (0.74 vs. 0.89 m) but similar between B_tin_ (0.92 m) and B (0.86 m) (*p* < 0.001, LSD = 0.026).

### Shoots and spikes

**Figure 7:**
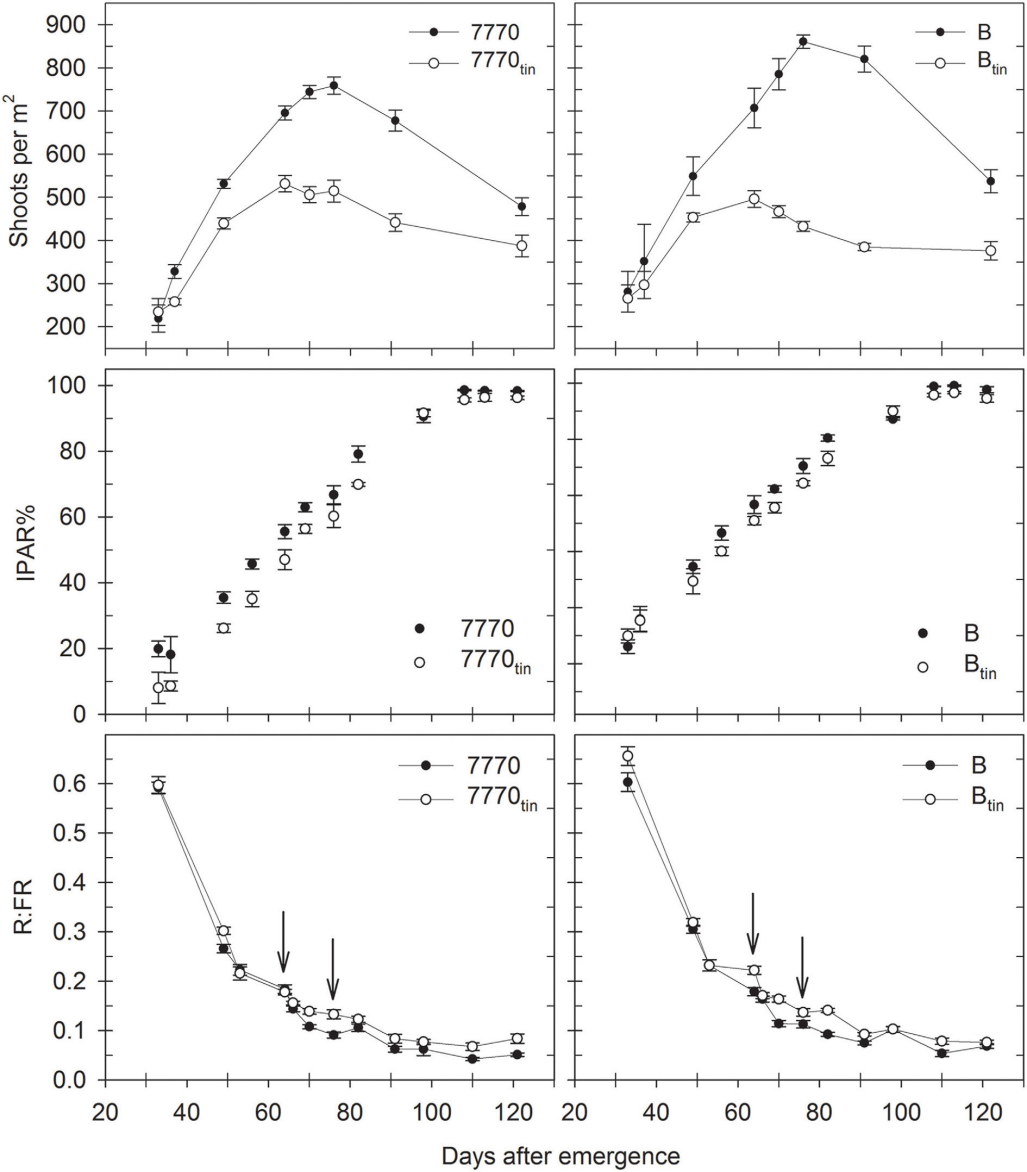
**Changes in shoot numbers, percentage of intercepted photosynthetic active radiation (IPAR%), and red: far-red ratio (R:FR) in two pairs of near-isogenic lines differing in the *tin* gene (7770/7770_tin_ and B/B_tin_) grown at Canberra in 2013**. Arrows indicate R:FR at maximum tiller number in *tin* (64 days after emergence) and free-tillering lines (76 days after emergence). Error bars show ± one standard error.

**Figure 8:**
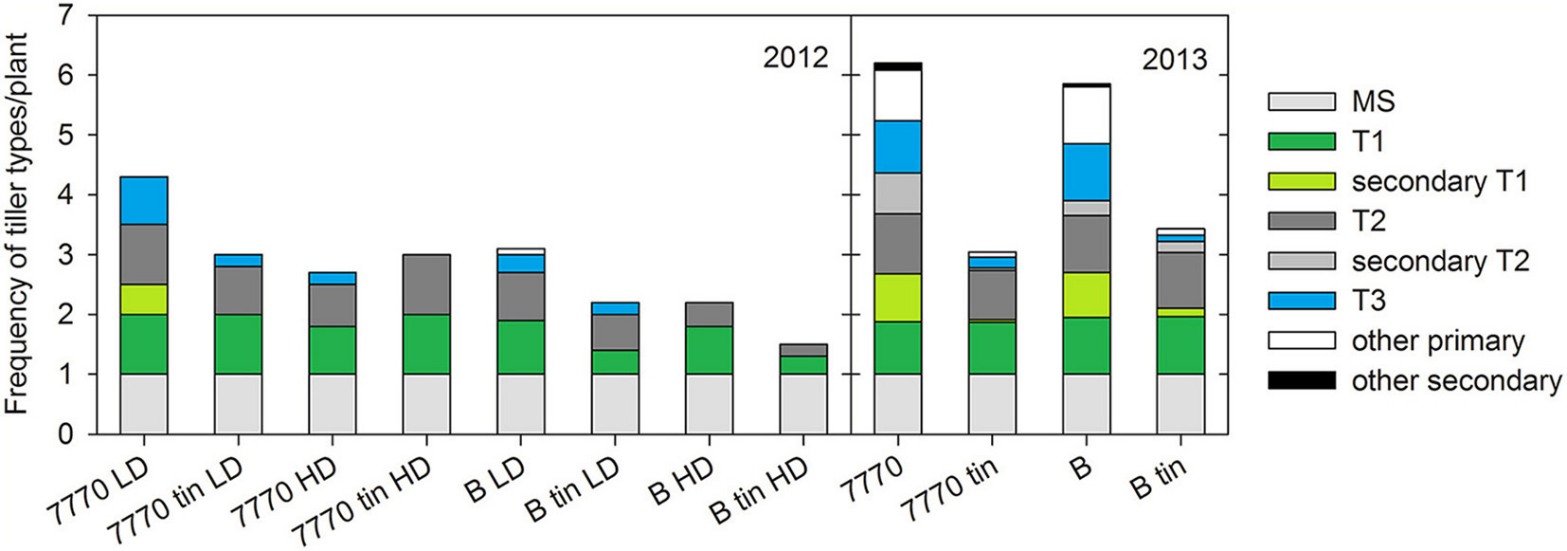
**Frequency of tiller types per plant in two pairs of near-isogenic lines differing in the *tin* gene (7770/7770_tin_ and B/B_tin_) grown at Canberra at a high (HD) and low (LD) plant density in 2012, and one uniform density in 2013: main shoot (MS), 1st primary tiller (T1) and secondary tillers on T1, 2nd primary tiller (T2) and secondary tillers on T2, 3rd primary tiller (T3), and other primary and secondary tillers**.

Overall, the *tin* lines produced fewer shoots and spikes compared to their free-tillering sisters in both seasons (Figures 7, 8). In 2012, the target plant densities were closely realized with 242 plants/m^2^ in the HD, and 141 plants/m^2^ in the LD treatment (*p* < 0.001, LSD = 11). Across densities, plant establishment was 192–196 plants/m^2^ except in line B_tin_ were early establishment was 171 plants/m^2^ (*p* < 0.001, LSD = 17). The effect of density carried through until maturity and was significant for the number of shoots counted at 54 DAE, corresponding with the expansion of flag leaves (504 shoots/m^2^ in the HD, and 415 shoots/m^2^ in the LD treatment; *p* = 0.023, LSD = 65), and spike number (123 spikes/m^2^ in the HD, and 95 spikes/m^2^ in the LD treatment; *p* < 0.001, LSD = 10.4). Around 30–50% fewer spikes than plants at establishment indicated that a number of plants died or did not produce a spike in this short and water-limited season. There was a significant effect of genotype on shoot (*p* = 0.006, LSD = 127) and spike number (*p* < 0.001, LSD = 11). At 54 DAE, the *tin* lines had 30–35% fewer shoots, i.e., 7770 and 7770_tin_ had 501 and 363 shoots/m^2^, and B and B_tin_ had 514 and 338 shoots/m^2^, respectively. Spike number was reduced by 16% in line 7770_tin_ compared to 7770 (106 vs. 127 spikes/m^2^) and by 40% in B_tin_ compared to B (76 vs. 126 spikes/m^2^).

In 2013, when plant establishment was exactly 125 plants/m^2^, the maximum shoot number was 30% less in line 7770_tin_ compared to 7770 (531 vs. 759 shoots/m^2^), and 40% less in line B_tin_ compared to B (494 vs. 861 shoots/m^2^) (*p* < 0.001, LSD = 62). Tillering ceased about 12 days earlier in the *tin* lines compared to the free-tillering sisters with the maximum number of shoots being measured at 64 DAE (08/08) in the *tin*, and at 76 DAE (20/08) in the free-tillering lines (Figure 7). At maturity, spike number was 20% reduced in line 7770_tin_ compared to 7770 (387 vs. 478 spikes/m^2^) and 30% reduced in B_tin_ compared to B (376 vs. 537 spikes/m^2^) (*p* < 0.001, LSD = 69). Continuous monitoring of changes in shoots numbers over time (Figure 7) allowed for the calculation of the tiller economy, which was significantly greater in the *tin* lines (7770_tin_: 0.73; B_tin_: 0.76) than in the free-tillering lines (7770: 0.63; B: 0.62), i.e., more tillers became reproductive shoots in the *tin* lines.

On a per plant basis, reduced tillering plasticity of *tin* genotypes was indicated by lower frequencies of later primary tillers (e.g., T3, T4) and secondary tillers (e.g., T1.1, T2.1), which ultimately resulted in lower numbers of tillers per plant in comparison to the free-tillering lines, especially under favorable growth conditions in 2013 (Figure 8). Note that the shoot numbers per plant derived from Figure 8 and Figure 7 vary slightly due to the sampling differences described above. Generally, primary tillers T1 and T2 were observed most frequently followed by T3 (Figure 8). The coleoptile tiller (T0) was observed almost exclusively in 2013 with a mean frequency of 0.2 in free-tillering and 0.06 in *tin* lines (data not shown). In 2012, all lines responded to increased plant density by reducing the numbers of later tillers. There were genotypic differences in the presence of T1 and T2 (*p* = 0.014), while the presence of T3 was influenced by both genotype (*p* = 0.011) and plant density (*p* < 0.001). Across densities, lines 7770 and 7770_tin_ produced on average about one T1 and 0.85 T2, line B had 0.85 T1 and 0.6 T2, and B_tin_ produced 0.37 T1 and T2 per plant. Overall, T3 appeared more frequently in the free-tillering compared to the *tin* lines. At HD, tiller T3 was either absent, as in line B and both *tin* genotypes, or present in small numbers as in line 7770 (0.18 vs. 0.84 at LD) (Figure 8). In 2013, all lines had an average of ~one T1 and T2, and genotypic differences were only evident in the numbers of secondary and higher order primary tillers (Figure 8). The free-tillering lines had on average around 0.9 T3 and the *tin* lines 0.15 T3 per plant (*p* < 0.001). Tillers T4 and T5 were mostly absent in the *tin* lines while the free-tillering lines produced about 0.5 T4 and 0.15 T5 (*p* < 0.001).

### Light interception, red: far-red ratio, and chlorophyll content

**Figure 9:**
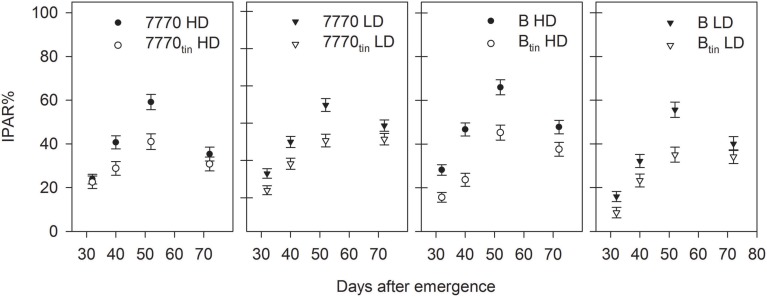
**Percentage of total intercepted photosynthetic active radiation (IPAR%) in two pairs of near-isogenic lines contrasting for the *tin* gene (7770/7770_tin_ and B/B_tin_) grown at high (HD) and low (LD) plant density at Canberra in 2012**. Error bars show ± one standard error.

The *tin* lines intercepted less radiation compared to the free-tillering lines, and this difference was generally significant throughout both seasons (*p* ≤ 0.05) (Figures 7, 9). In 2012, the effect of plant density on IPAR% was mostly significant (*p* < 0.001). The IPAR% was greater at HD than at LD, expect for the last measurement taken during seed-filling (72 DAE). The decline of IPAR% at 72 DAE in all treatment combinations can be explained by the collapsing of leaves in the rapidly senescing, water-stressed crop (Figure 9). Differences in canopy development between NILs were associated with consistently lower light interception and groundcover (GC%) in *tin* lines compared to the free-tillering lines. For example, lines 7770 and 7770_tin_ intercepted 19% and 14% IPAR%, and lines B and B_tin_ intercepted 22 and 12% IPAR% during tillering at 32 DAE (*p* = 0.004, LSD = 4.6). At this stage, GC% was 33 and 22% in the *tin* lines 7770_tin_ and B_tin_ but around 40% in the free-tillering sisters (*p* < 0.001, LSD = 3.6).

Under favorable growth conditions in 2013, light interception was reduced in *tin* lines compared to the free-tillering sisters from late tillering until maximum canopy expansion. Thus, light penetrated deeper into the canopies of *tin* lines and hence R:FR measured at the plant base was generally greater in *tin* compared to non-*tin* lines (Figure 7). At 70 DAE during tillering, IPAR% was 56% in the *tin* lines and 62–63% in the free-tillering sisters (*p* < 0.001, LSD = 4.1). Reduced light interception in *tin* lines can be explained by their smaller canopies as indicated by fewer tillers (Figure 7) and reduced GC%, which was 73–74% in the *tin* lines and 81–82% in the free-tillering lines during tillering at 70 DAE (*p* < 0.001, LSD = 0.9). At 106 DAE, when flag leaves were fully expanded, the IPAR% was 96% in the *tin* lines and 99% the free-tillering lines (*p* < 0.001, LSD = 1.3) (Figure 7).

The maximum tiller numbers were observed at 64 DAE in the *tin* lines and at 76 DAE in the free-tillering lines (Figure 7). At this stage, both R:FR and PAR% measured at ground-level were greater in the *tin* lines compared to the free-tillering sisters. The R:FR ratio at maximum tiller number was 0.18 and 0.09 in lines 7770_tin_ and 7770, and 0.22 and 0.11 in lines B_tin_ and B (*p* < 0.001, LSD = 0.02), and PAR% was 53% and 33% in lines 7770_tin_ and 7770, and 49 and 30% in lines B_tin_ and B, respectively (*p* < 0.001, LSD = 8).

Generally, chlorophyll (Chl) measurements showed that the *tin* lines maintained the green area of lower leaves for longer into the season. In 2012, the Chl estimated at about 1 week before anthesis (53 DAE) on fully grown flag leaves and subjacent 4th leaves was greater at LD compared to HD and greater in the *tin* compared to the free-tillering lines (*p* < 0.001). Across lines, the flag leaf Chl was 56.8 (SPAD units) at LD and 53.5 at HD (LSD = 2.3), and Chl of the 4th leaf from top was 50.3 at LD and 44.4 at HD (LSD = 3.7). The Chl of flag leaves of lines 7770 and 7770_tin_ was 56.8 and 59, and that of lines B and B_tin_ was 51.6 and 53 (LSD = 1.6). The 4th leaf from top had a Chl of 43.7 and 46.2 in lines 7770 and 7770_tin_, and 43.8 and 55.8 in lines B and B_tin_ (LSD = 2.6).

**Figure 10:**
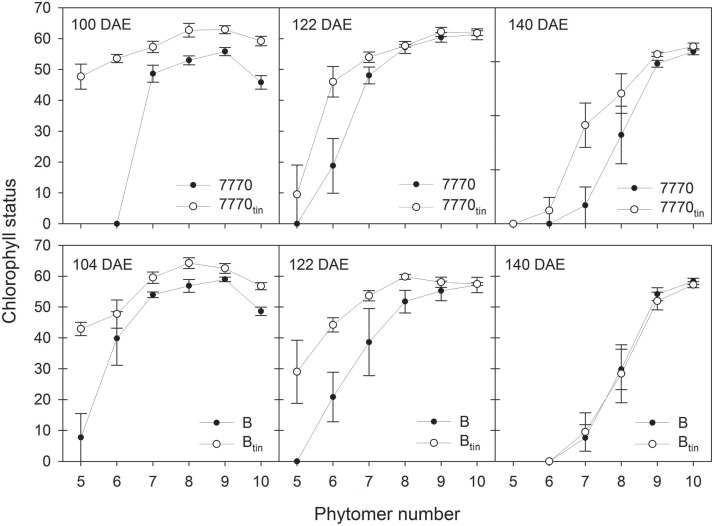
**Chlorophyll status (SPAD units) of main shoot leaves at mid to late booting stage (100–104 days after sowing; DAE), around early anthesis (122 DAE), and seed filling (140 DAE) in two pairs of near-isogenic lines differing in the *tin* gene (7770/7770_tin_ and B/B_tin_) grown at Canberra in 2013**. Phytomer 10 corresponds to the flag leaf. Error bars show ± one standard error.

In 2013, changes in chlorophyll content with leaf age were monitored from full expansion of flag leaves (mid/late booting) until seed-filling (Figure 10). The flag leaves of *tin* lines had initially significantly higher Chl values (*p* < 0.001, LSD = 4.9) but this difference observed at 100–104 DAE (booting) disappeared as the flag leaves aged (Figure 10). Lower in the canopy, the Chl of the 6th leaf of the main shoot (phytomer six) of the *tin* lines was greater at 100–104 DAE (*p* < 0.001, LSD = 19) and 122 DAE (*p* = 0.024, LSD = 21.5) but leaf 6 had senesced at 140 DAE (low or zero Chl). At the start of flowering (122 DAE), the senescence of lower leaves was more advanced in the free-tillering lines compared to the *tin* lines (Figure 10). During seed-filling at 140 DAE, the Chl of leaves of *tin* and free-tillering lines were mostly similar except the Chl of leaf seven, which was greater in line 7770_tin_ compared to 7770 (*p* = 0.006, LSD = 18.4). The leaf Chl of the primary tillers T1 and T2 resembled those of the main shoot (not shown).

### Vertical distribution of leaf area and light

The free-tillering lines produced greater total leaf area than the *tin* lines. In 2012, the anthesis LAIs (Z60–69; measured at 65 DAE) were 0.75 and 0.57 in lines 7770 and 7770_tin_, and 0.56 and 0.4 in lines B and B_tin_ (*p* < 0.001, LSD = 0.13; data not shown). Senescence was advanced at this stage, which contributed to the anthesis LAIs being low in 2012. In 2013, the LAIs at the start of spike emergence (Z50–52; LAIs measured between 106 and 117 DAE) were 6.53 and 5.58 in lines 7770 and 7770_tin_, and 9.26 and 5.6 in lines B and B_tin_, respectively (*p* = 0.008, LSD = 2.1). The LAIs declined subsequently due to senescence, and were statistically similar toward the end of anthesis (Z69–70; LAIs measured between 132 and 135 DAE) with a grand mean LAI of 4.2 (*p* = 0.15).

**Figure 11:**
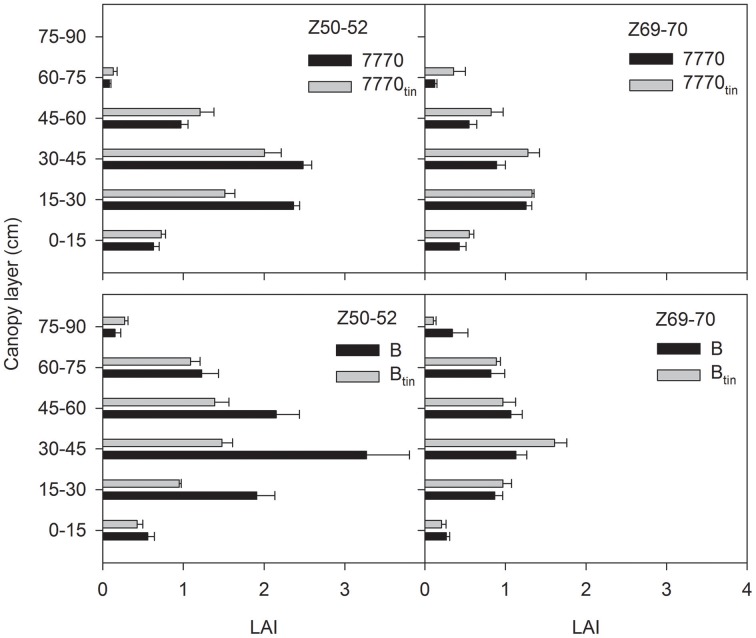
**Distribution of green leaf area index (LAI) with canopy height at the start of spike emergence (Z50-52) and late anthesis (Z69-70) in two pairs of near-isogenic lines differing in the *tin* gene (7770/7770_tin_ and B/B_tin_) grown at Canberra in 2013**. Error bars show one standard error.

The vertical distribution of green leaf area differed between *tin* and non-*tin* lines, and this changed with senescence (Figure 11). At the start of spike emergence (Z50–52), the green leaf area at lower to mid-height of the canopy (0.15 to 0.45 m) was significantly greater in the free-tillering compared to the *tin* lines (*p* < 0.02). However, toward the end of anthesis (Z69–70) the leaf area in the 0.3–0.45 m canopy layer was greater in *tin* lines (*p* = 0.02) but similar for the sister-lines in the 0.15–0.3 m layer (*p* = 0.1).

**Figure 12:**
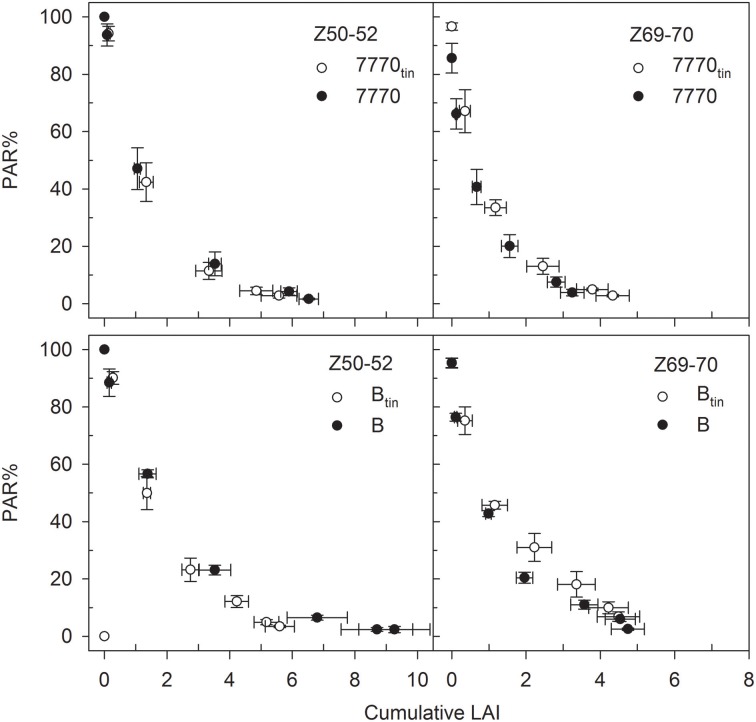
**Changes in the percentage photosynthetic active radiation (PAR%) with canopy depth as indicated by the cumulative leaf area index (LAI) at the start of spike emergence (Z50-52) and late anthesis (Z69-70) in two pairs of near- isogenic lines differing in the *tin* gene (7770/7770_tin_ and B/B_tin_) at Canberra, 2013**. The data pair PAR% = 100 and LAI = 0 corresponds to the measurements taken above the canopy, and the lowest PAR% and greatest LAIs were measured at ground-level. Bi-directional error bars show ± one standard error.

The amount of PAR% transmitted by the canopy declined exponentially with canopy depth (Figure 12). The light attenuation characteristics of the *tin* lines differed from those of the free-tillering lines in that the light extinction coefficient *k* was greater in the *tin* lines at the start spike emergence (7770_tin_: 0.64; 7770: 0.59; B_tin_: 0.57; B: 0.41; r^2^ > 0.98 and RMSD < 3.2 for all exponential regressions), and lower in the *tin* lines compared to the free-tillering lines at the end of anthesis (7770_tin_: 0.82; 7770: 0.98; B_tin_: 0.56; B: 0.69; r^2^ > 0.95 and RMSD < 11.2 for all exponential regressions).

## Discussion

Using specifically developed NILs contrasting for the *t*iller *in*hibition gene, *tin*, this study demonstrated that *tin* modifies different, seemingly genetically-unrelated, canopy architectural attributes such as the maximum number of shoots per plant and the distribution of organ sizes along shoots. Relationships between architecture and physiological functions observed in NILs contrasting for *tin* are discussed herein.

### Plasticity in bud break

Tillering in the *tin* lines ceased at a greater R:FR ratio than in the free-tillering lines, and this was independent of the observation that plant bases of *tin* lines received more radiation and were exposed to greater R:FR than the free-tillering sisters over much of the growing season. Later tiller buds in *tin* lines can break dormancy to initiate and grow though at reduced frequencies (Figure 8) indicating that there is some plasticity for higher-order buds in response to environmental cues in the *tin* lines. However, it is still unclear to what extent such reduced levels of tillering plasticity in *tin* genotypes are mediated by light quality and especially R:FR signal transduction through the different phytochromes present in higher plants (Franklin and Whitelam, 2005). It will be interesting to further evaluate the tillering plasticity of *tin* genotypes in light and R:FR environments generated by the growing of plants on regular grids of different plant densities (Evers et al., 2006) or by manipulating light levels and/or R:FR artificially (Mandoli and Briggs, 1981; Casal et al., 1990) to explore the idea of a defined, genotype-specific R:FR threshold at which tillering ceases to be implemented in models of crop growth and development (Evers and Vos, 2013).

### The relation between bud break and internode elongation

Elongating internodes are strong sinks for photo-assimilates, and Kebrom et al. (2012) hypothesized that precocious internode development in *tin*-containing lines contributes to the early arrest of tiller bud growth through sucrose starvation. Similar to the controlled glasshouse study by Kebrom et al. (2012), we observed under field conditions that the basal internodes of the *tin* lines elongated at lower phytomer ranks compared to the free-tillering sisters, though this was more pronounced in the Banks-derived line B_tin_ (a sister-line of B_tin_ was evaluated by Kebrom et al., 2012) than in the Wyalkatchem-derived 7770_tin_. However, there was also environmental plasticity as internode elongation started at a lower phytomer rank in 2012 than in 2013 (Figure 6). Such plasticity has been previously observed in wheat where increased sowing depth and subsequently crown depth was associated with basal internodes elongating earlier, i.e., at a lower phytomer rank (Kirby, 1993).

Up until now it is unclear if the greater R:FR ratio at which tillering ceased in *tin* genotypes, as discussed above, is indicative of an active process mediated by phytochrome or by resource limitation as suggested by Kebrom et al. (2012). The “nutritional hypothesis” (Assuero and Tognetti, 2010) for an early arrest of tiller bud growth is supported by the observation of low levels of sucrose in tiller buds that did not grow (Kebrom et al., 2012), and the recent finding that apical dominance in pea (*Pisum sativum* L.) was predominantly controlled by the strong demand of the shoot tip for sugars, which inhibited axillary bud outgrowth (Mason et al., 2014). Alternatively, genetic interaction between *tin* and genes responsible for photomorphogenic responses (Franklin and Whitelam, 2005) might modify the sensitivity to R:FR and consequently the photomorphogenesis of *tin*-containing lines compared to free-tillering lines. Map-based cloning of the *tin* gene is currently underway to identify the gene and physiological mechanism contributing to the observed variation in tillering response of *tin*-containing lines (W. Spielmeyer, personal communications).

### Green leaf area in lower canopy

It has been shown that canopy light gradients are closely related to the vertical distribution of N per unit leaf area in wheat canopies (Bertheloot et al., 2008, 2012). Both *tin* lines maintained the green area of leaves in the lower canopy, and therefore leaf N content (Debaeke et al., 2006), for longer into the season. Prior to flowering, leaf N contents were greater in the *tin* lines (Figure 10). There is a strong relationship between leaf N content and the maximum (light-saturated) photosynthetic rate (Evans, 1989; Dreccer et al., 1998), which may have been greater in the *tin* lines just before flowering. Greater light penetration in the canopies of *tin* lines would have contributed to delaying senescence in the lower canopy (Figures 7, 9), and can improve radiation-use efficiency (Dreccer et al., 1998). Another factor for maintaining the leaf area in the lower canopy would be the improved availability of water and N per reproductive shoot associated with reduced tiller numbers and greater tiller economy. The greater leaf N contents of the upper-most leaves in *tin* lines observed prior to flowering may indicate increased availability of water and N per reproductive shoot rather than more available light. As the developing inflorescence represents a strong sink for N and carbon, improved tiller economy (Figure 7) and subsequent maintenance of leaf N content in the lower canopy (Figure 10) could be important traits for improving seed quality (protein content and seed size).

### Light extinction

Reduced light interception in the *tin* genotypes at maximum leaf area expansion, which typically occurs before anthesis when flag leaves are fully expanded (Moeller et al., 2007; Foulkes et al., 2001), can be explained by their smaller canopy size as associated with lower numbers of shoots and smaller LAIs. However, light interception would have been also influenced by the geometric and optical properties of the contrasting canopies as indicated by the different light extinction coefficients (*k*) calculated for the NILs. Variation in *k* for NILs contrasting in *tin* may be expected given the observed differences in distributions of organ sizes (Figures 4–6) and leaf area (Figure 11). Any interpretations of *k* are notoriously difficult because the geometric and optical properties of canopies entail many interacting attributes (e.g., leaf size distribution, leaf angle, orientation, curvature as well as light reflectance and transmittance) changing dynamically in time and space, and some may even compensate for each other. The extinction coefficient *k* changes concurringly: prior to spike emergence, we observed lower *k*-values in the smaller canopies of the *tin* lines compared to higher *k*-values in the larger canopies of the free-tillering lines while this was reversed after spike emergence. In contrast, Foulkes et al. (2001) speculated that small canopies may be “predisposed to have higher *k*, through having thicker, less transmissible leaves” (p. 14). Green (1987) expected “a continual decline of *k* with age” (p. 219) due to ontogenic increases in leaf angle (i.e., downward movement of leaves). This contradicts our observation in which *k* increased from prior to spike emergence until the end of spike emergence in all but one line (B_tin_). This increase would be related to light interception by spikes as well changes in green area with plant age. Thus, *k* is mainly useful to show that there are differences in optical properties but to dissect and further understand any sources of variation in *k* in NILs contrasting for *tin* requires a more integrated approach that explicitly takes into account the spatial distribution of plant organs. The most feasible approach for achieving this is arguably spatially-explicit modeling of plant architecture, growth, development and physiological functioning called functional-structural plant (FSP) modeling (Vos et al., 2010; Evers and Vos, 2013). Based on existing FSP models of wheat (Evers et al., 2005), contrasting seasonal time-courses of tiller production and mortality as well as leaf area expansion and arrangement could be simulated to explore (i) the relationship with cumulative radiation interception, which is important for potentially achieving high productivity (Monteith, 1977), and (ii) the possible consequences for the timing of transpirational water-use relative to anthesis and seed-filling, which is important for crop adaptation to water-limited environments (Passioura and Angus, 2010; Rebetzke et al., 2013).

## Conclusion

This is the first study to quantify canopy architectural differences of NILs differing in the *t*iller *in*hibition gene *tin*. Using two contrasting genetic backgrounds grown in contrasting field environments, the study showed that the *tin* gene modifies the canopy architecture of wheat by increasing tiller economy and changing the distribution of organ sizes thereby varying the time course of resource-use. Associated with the early cessation of tillering in *tin* lines are reduced probabilities for later primary and higher-order tillers. The *tin*-containing genotypes intercepted less radiation as a consequence of reduced leaf area as well as differences in the vertical distribution of leaf area. The *tin* genotypes maintained the green area of leaves in the lower canopy for longer and this is likely to be related to lower light gradients and increased water and N availability per reproductive shoot. More radiation penetrated at canopy depth in *tin* lines, which could improve radiation-use efficiency. Spatially-explicit FSP modeling of canopy architecture, growth, and development is arguably the most feasible means to further elicit details of the relationships between earlier vs. later cessation of tillering in response to different R:FR signals, the evolving and expanding canopy structures post tillering, and the consequent light attenuation characteristics (*k*) of the contrasting canopies explored here.

### Conflict of interest statement

The Associate Editor Sergey Shabala and the Reviewer Colin James Birch declare that, despite being affiliated to the same institution as the author Carina Moeller, the review process was handled objectively. The authors declare that the research was conducted in the absence of any commercial or financial relationships that could be construed as a potential conflict of interest.
